# Glycemic control and clinical outcomes in patients with diabetes and atrial fibrillation: a nationwide cohort study

**DOI:** 10.1007/s00592-026-02725-1

**Published:** 2026-06-22

**Authors:** Elis Kouki, Birgitta Salmela, Aapo Aro, Olli Halminen, Konsta Teppo, Leo Niskanen, Jari Haukka, Jukka Putaala, Miika Linna, Pirjo Mustonen, Juha Hartikainen, K. E. Juhani Airaksinen, Mika Lehto

**Affiliations:** 1 University of Helsinki, 00270 Helsinki, Finland; 2 Department of Internal Medicine, Heart Center, Päijät-Häme Central Hospital, Lahti, Finland; 3 Heart and Lung Center, Helsinki University Hospital, and University of Helsinki, Helsinki, Finland; 4 University of Eastern Finland, Kuopio, Finland; 5 Heart Centre, Turku University Hospital, and University of Turku, Turku, Finland; 6 Department of Internal Medicine, Päijät-Häme Central Hospital, Lahti, Finland; 7 Department of Neurology, Helsinki University Hospital, and University of Helsinki, Helsinki, Finland; 8 University of Eastern Finland, Kuopio, Finland; 9 Aalto University, Espoo, Finland; 10 Turku University Hospital, and University of Turku, Turku, Finland; 11 Kuopio University Hospital, and University of Eastern, Kuopio, Finland; 12 Jorvi Hospital, Department of Internal Medicine, HUS Helsinki University Hospital, and University of Helsinki, Helsinki, Finland

**Keywords:** Atrial fibrillation, Diabetes mellitus, Glycated hemoglobin, Mortality, Ischemic stroke, Myocardial infarction

## Abstract

**Aims:**

To evaluate the association of HbA1c level at the time of atrial fibrillation (AF) diagnosis with adverse outcomes (death, ischemic stroke (IS), myocardial infarction (MI), bleeding) in an unselected nationwide cohort of patients with AF and diabetes mellitus (DM).

**Methods:**

The retrospective FinACAF registry study covered all patients with incident AF in Finland between 2010 and 2017. Outcomes were analyzed using HRs and sHRs (hazard ratios and subdistribution hazard ratios), with HbA1c modelled both categorically and continuously.

**Results:**

Among 157 658 patients with incident AF, DM was present in 23% (n = 35 872). Baseline HbA1c was available in 49% (17 519) of DM patients. DM was associated with increased hazards of all evaluated outcomes. Elevated HbA1c in categorical analyses was associated with progressively increased adjusted HRs and sHRs, with highest ratios seen in HbA1c > 63 mmol/mol for death (HR 1.56, 95% CI 1.45–1.68), IS (sHR 1.38 95% CI 1.20–1.59), and MI (sHR 1.70 95% CI 1.48–1.94), when compared to patients without DM. In continuous analyses, higher HbA1c levels were associated with increasing hazards for mortality, IS, and MI. Bleeding hazards remained stable across the HbA1c spectrum.

**Conclusions:**

Higher baseline HbA1c levels were associated with progressively increased hazards of mortality, IS, and MI in patients with incident AF and DM.

**Supplementary Information:**

The online version contains supplementary material available at 10.1007/s00592-026-02725-1.

## Introduction

Atrial fibrillation (AF) is the most prevalent sustained cardiac arrhythmia, affecting up to 5.2% of adults [1]. AF is associated with increased mortality, and prognosis is largely influenced by the risk of stroke, heart failure, and the presence of comorbidities, making comprehensive care and risk factor modification essential for improving the outcomes of these patients [2, 3].

Diabetes mellitus (DM) is one of the most prevalent chronic diseases and represents a major cardiovascular risk factor, including in the development of AF [4]. The presence of DM is associated with an increased risk of AF-related adverse outcomes [5–7]. In addition, poor glycemic control in individuals with AF and DM seems to be associated with higher rates of stroke and increased mortality [8–10]. Thus, effective glycemic control is recommended as part of comprehensive risk factor management to mitigate overall cardiovascular risk and reduce the incidence of stroke in patients with AF and DM [3].

The prevalence and incidence of both AF and type 2 DM are increasing, as is the prevalence of DM among individuals with AF [5]. This overlap poses a growing public health concern and highlights the importance of investigating the prognostic implications of their coexistence [3].

This nationwide retrospective registry study aimed to assess the associations between HbA1c levels at the time of AF diagnosis and adverse events (death, ischemic stroke (IS), myocardial infarction (MI), and bleeding) in an unselected cohort of patients with AF and DM. This study leverages a comprehensive nationwide cohort capturing AF patients across all levels of care [11].

## Materials and methods

### Study population

This substudy is part of the Finnish AntiCoagulation in Atrial Fibrillation study (FinACAF) (ClinicalTrials Identifier: NCT04645537; ENCePP Identifier: EUPAS29845), a retrospective nationwide registry-based cohort study gathering information on all Finnish AF patients between 2004 and 2018. A detailed description of the study methods has been published previously [11]. This substudy includes patients over 20 years of age whose first-ever International Classification of Diseases, Tenth Revision, (ICD-10) diagnosis code of I48 was recorded between January 1st 2010 and December 31st 2017.

AF patients were identified from all national health care registries: Hilmo (hospitalizations and outpatient specialist visits), AvoHilmo (primary health care visits), and the National Reimbursement Registry for reimbursed medication upheld by the Social Insurance Institute (KELA). To ensure a cohort of only incident AF we excluded patients who received warfarin prescriptions from 2004–2006 due to their short recorded medical history and high likelihood of having prior AF. We also excluded patients with recorded use of any oral anticoagulant (OAC) up to one year before cohort entry. Supplementary Figure SF1 shows the patient selection flow chart.

Individual patient data from all the used registries were linked via a unique personal identification number given to every Finnish citizen.

Patients were classified as having DM if they had a recorded DM diagnosis code (ICD-10: E10-E14) in the hospital or primary care registry, a reimbursed DM medication in the National Reimbursement Registry or had redeemed DM medications before their AF diagnosis. Patients with none of the above criteria but with a baseline HbA1c of ≥ 48 mmol/mol (6.5%) were also classified as having DM (n = 477). This criterion aligns with the Finnish Current Care Guidelines, whereby a single HbA1c measurement ≥ 48 mmol/mol suffices for the diagnosis of DM in Finland [12]. Additionally, a comprehensive review of laboratory data from 2010 onward confirmed that the majority of these patients (n = 327) had repeated HbA1c measurements meeting this threshold.

### Baseline variables

The baseline variables were gathered from January 1.st 2004 up to the cohort entry date. The baseline value for blood HbA1c was defined as the mean of all HbA1c measurements taken within the year prior to AF diagnosis. The definitions and details of the baseline variables are presented in Supplementary Table ST1. The laboratory databases of the six largest central laboratories in Finland were used to obtain the HbA1c values. Laboratory data were available for a catchment population representing 77% of the Finnish population [11].

### Outcomes

The outcomes analyzed were all-cause death, IS, MI, and any bleeding after the AF diagnosis. The ICD-10 codes used to define the outcomes have been previously established and are detailed in Supplementary Table ST2 [11]**.** In patients without prior events of interest IS, MI or bleeding were considered to have occurred on the first date of a recorded ICD-10 diagnosis code in the hospital care registry after cohort entry. In patients with prior events of interest, a recurrent event was considered to have occurred on the date of the first new hospitalization, with at least a 90-day gap from the prior event that had occurred before AF diagnosis.

Dates of death were retrieved from the National Causes of Death Registry upheld by Statistics Finland.

### Study ethics

Ethical permission was granted by the ethical committee of the Medical Faculty of Helsinki University, Helsinki, Finland, and study permission by the Helsinki University Hospital. Respective permissions were obtained from the Finnish registry holders (KELA 138/522/2018; THL 2101/5.05.00/2018; Population Register Centre VRK/1291/2019–3; Statistics Finland [TK-53–1713-18/u1281]; Tax Registry VH/874/07.01.03/2019). The patient data were pseudonymized by KELA, providing unidentifiable but individualized patient data to the research group.

### Statistical analysis

Statistical analyses were performed with the IBM SPSS Statistics for Windows software (V.28.0, IBM) and R statistical software (V.4.2.2, R Foundation for Statistical Computing, Vienna, Austria. URL: https://www.R-project.org/).

Follow-up time was calculated from cohort entry until the date of the respective outcome of interest, death, or 31.12.2018, whichever occurred first. To account for aging and temporal trends, we utilized Lexis expansion to split follow-up time into 1-year intervals while age was time-updated into 5-year intervals [13]. Age and calendar year were treated as categorical variables.

Baseline variables were compared between diabetic patients with and without an available baseline HbA1c using standardized mean differences (SMD), with an SMD > 0.10 indicating a meaningful imbalance (Supplementary Table ST3**)**. To further address selection bias regarding missing baseline HbA1c we utilized stabilized inverse probability weighting (SIPW) when modelling HbA1c as a continuous variable. Propensity scores for having an observed HbA1c measurement were derived from a logistic regression model that included age, sex, income, education, baseline statin use, hypertension, hyperlipidemia, psychiatric disorders, dementia, cancer, vascular disease, alcohol use disorder, prior bleeding, prior IS, liver cirrhosis or failure, impaired renal function, and heart failure. Patients with an observed value were weighted to more accurately represent the entire diabetic cohort. Stabilized weights were chosen to preserve the effective sample size and prevent artificial variance inflation. The propensity score model demonstrated adequate overlap (c-statistic 0.56). The positivity assumption was satisfied (propensity scores ranged from 0.32 to 0.70), and the resulting stabilized weights exhibited a narrow distribution (mean 1.00, range 0.70–1.56), requiring no truncation. All evaluated baseline variables achieved a SMD < 0.10 post-weighting (Supplementary Table ST3). In the categorical analyses missing data were handled by creating a distinct “Diabetes, no HbA1c” stratum.

We evaluated the dose response association between HbA1c and clinical outcomes using natural cubic splines. Prespecified Internal knots were placed at the 10th, 50th, and 90th percentiles of the continuous HbA1c distribution (in accordance with established biostatistical guidelines [14]), with boundary knots positioned at the 5th and 95th percentiles to constrain variance at the extreme tails. To ensure robustness of the spline shape we conducted sensitivity analyses across all endpoints using alternative 5- and 4-knot placements (Supplementary figure SF2). The continuous hazard was modeled using a piecewise exponential survival model via quasi-Poisson regression on the time-split Lexis data, including an offset for the natural logarithm of person-time. This specific framework produces hazard ratios (HRs) practically equivalent to a Cox proportional hazard model [15]. The quasi-Poisson distribution also accounts for potential overdispersion via a dispersion parameter, scaling the standard errors and mitigating the risk of falsely narrow confidence intervals. Utilizing generalized linear models on discrete person-time data is an established methodology when incorporating weights in time-to-event analyses [16]**.** HRs and 95% confidence intervals (CIs) were calculated across the continuous HbA1c spectrum, with 48 mmol/mol defined as the reference value. The proportional hazard assumption for continuous HbA1c was verified across all endpoints by testing its interaction with follow-up time.

In categorical analyses patients were classified by DM status and stratified by HbA1c levels. Time-dependent Cox proportional hazard models were used to estimate adjusted HRs. For IS, MI and bleeding outcomes we further estimated subdistribution hazard ratios (sHR) utilizing Fine-Gray competing risk models, with all-cause mortality acting as the competing event. Due to the interpretation of time-dependent variables being mathematically problematic within a subdistribution framework, the Fine-Gray models were constructed using only static baseline variables for adjustment.

All adjusted HRs and sHRs accounted for age, sex, calendar year, income level (quartiles), education level (International Standard Classification of Education ISCED 0–1, ISCED 2–4, ISCED 5 or higher), baseline statin use and clinical comorbidities (hypertension, hyperlipidemia, psychiatric disorders, dementia, cancer, vascular disease, alcohol use disorder, prior bleeding events, prior stroke, liver cirrhosis or failure, impaired renal function, and heart failure). The time-dependent models were additionally adjusted for OAC use as a time-varying variable. OAC treatment initiation was marked by the first purchase, and continuation was assumed until 120 days after the last drug purchase. This 120-day interval accounts for the Finnish national restriction of dispensing a maximum 90-day supply of reimbursed medication per purchase, plus a 30-day grace period to cover potential stockpiling and differences in warfarin dosing.

Baseline variables were compared using the Chi-square test, Student’s T-test and analysis of variance.

### HbA1c stratification

HbA1c was stratified into four subgroups: HbA1c < 42 mmol/mol (equivalent HbA1c < 6%), ≥ 42 to < 48 mmol/mol (6–6.5%), ≥ 48 to ≤ 63 mmol/mol (6.5–8%), and > 63 mmol/mol. The upper three categories were prespecified based on established clinical parameters: 48 mmol/mol represents the diagnostic threshold for diabetes mellitus and is a common treatment target, while values above 63 mmol/mol (corresponding to an estimated average glucose of approximately 10.2 mmol/L) reflect chronic poor glycemic control [17]. Following visual inspection of the continuous spline analyses, the lowest subgroup (< 42 mmol/mol) was added post hoc.

## Results

### Study cohort and baseline variables

We identified 157 658 patients (49.9% female) with incident AF. The mean age of the cohort was 72.9 ± 13.1 years at the time of entry. DM was prevalent in 23% (35 872) of the population. Compared to those without DM, patients with DM were older (mean age 74.8 vs 72.3 years, p < 0.001) and more frequently male (50.6% vs. 47.7%, p < 0.001), with a higher overall burden of baseline comorbidities (Table 1 and Supplementary Table ST4). During a mean follow-up of 3.8 years, 47 798 (30.3%) of patients died, 10 074 (6.4%) suffered an IS, 7 845 (5.0%) an MI, and 20 391 (12.9%) a bleeding event.

**Table 1:** Baseline variables of the study cohort

	No DM	DM with HbA1c (n = 17 519, 11.1%) HbA1c (mmol/mol)			
		< 42	≥ 42 to < 48	≥ 48 to ≤ 63	> 63
*N (% of total cohort)*	121 786 (77.2)	4 329 (2.7)	4 829 (3.1)	5 900 (3.7)	2 461 (1.6)
*Demographics*					
Sex, Female*,**	50.6	46.2	49.5	47.5	44.2
Mean age, years (SD)*,**	72.3 (13.7)	73.9 (9.5)	75.2 (9.5)	75.7 (10.2)	73.1 (11.7)
*Comorbidities*					
Hypertension*	70.9	92.0	92.3	92.0	90.8
Heart failure*,**	15.3	18.7	20.3	27.5	33.2
Hyperlipidemia*, **	42.9	74.6	76.6	77.2	77.2
Ischemic stroke*,**	10.8	12.3	13.1	14.9	17.1
Transient ischemic attack (TIA)	4.3	5.0	4.5	4.5	4.0
Vascular disease*,**	24.8	34.0	39.0	44.6	49.1
Coronary artery disease*,**	19.9	27.6	31.7	37.2	40.0
Myocardial infarction*,**	7.7	9.2	11.5	14.3	17.5
Prior bleeding*, **	10.4	14.3	13.1	14.7	15.3
Liver cirrhosis or failure*, **	0.4	1.8	0.6	1.0	1.1
Impaired renal function*,**	3.1	7.5	6.4	9.6	13.8
*Risk scores*					
Mean CHA2DS2-VA score (SD)*,**	2.6 (1.6)	4.1 (1.5)	4.2 (1.4)	4.4 (1.5)	4.4 (1.6)
Mean modified HAS-BLED score (SD)*	2.0 (1.1)	2.5 (1.0)	2.5 (1.0)	2.6 (1.0)	2.5 (1.1)
*Medication*					
Statin*, **	29.9	58.9	62.0	62.7	62.3
Other lipid lowering medication*	1.1	2.6	2.9	3.0	3.1

CHA2DS2-VA (congestive heart failure, hypertension, age ≥ 75 years, diabetes, history of stroke/TIA, vascular disease, age 65–74 years). HAS-BLED (hypertension, abnormal renal or liver function, prior stroke, bleeding history, age > 65 years, alcohol abuse, concomitant antiplatelet/nonsteroidal anti-inflammatory drugs (max score 8 as labile INR was unavailable)

*DM* diabetes mellitus

*p < 0.05 DM vs. Non-DM; **p < 0.05 within DM HbA1c subgroups

Among patients with DM, 49% (17 519) had at least one HbA1c measurement recorded within the year prior to AF diagnosis. A higher HbA1c in patients with DM associated with a higher prevalence of heart failure, previous IS and MI, vascular disease, sleep apnea, dementia, and impaired renal function (p < 0.05). Further baseline variables are presented in Supplementary Table ST4.

### Missing HbA1c data

Baseline variables of patients with DM with and without available HbA1c measurements are described and compared in Supplementary Table ST3. SMDs revealed that patients with available HbA1c data exhibited higher rates of diagnosed hyperlipidemia (76.4% vs 70.7%, SMD = 0.129) and baseline statin use (61.5% vs 53.3%, SMD = 0.167).

### Outcomes

**Fig. 1:**
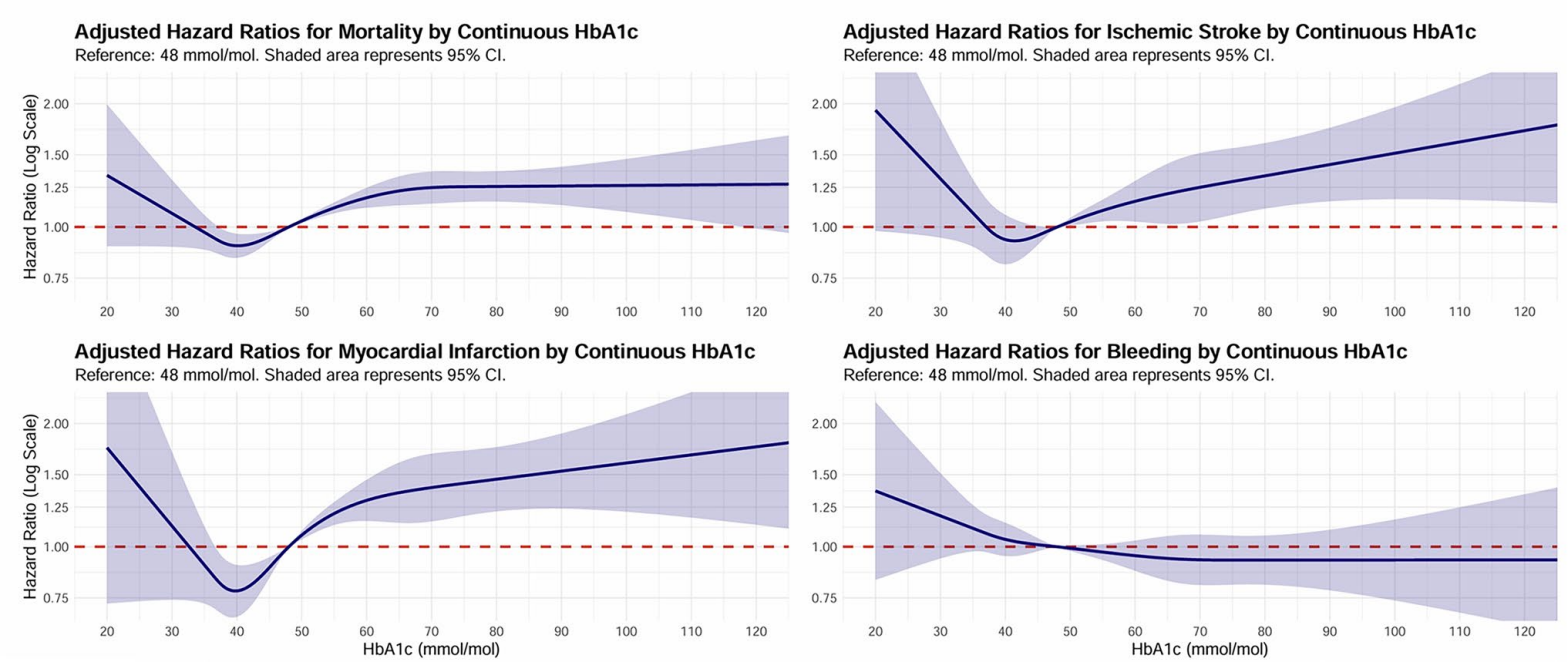
Adjusted hazard ratios for evaluated outcomes by continuous HbA1c in patients with atrial fibrillation and diabetes mellitus (Note: confidence intervals substantially widen at the upper and lower extremes of the HbA1c distribution due to sparse data)

**Fig. 2:**
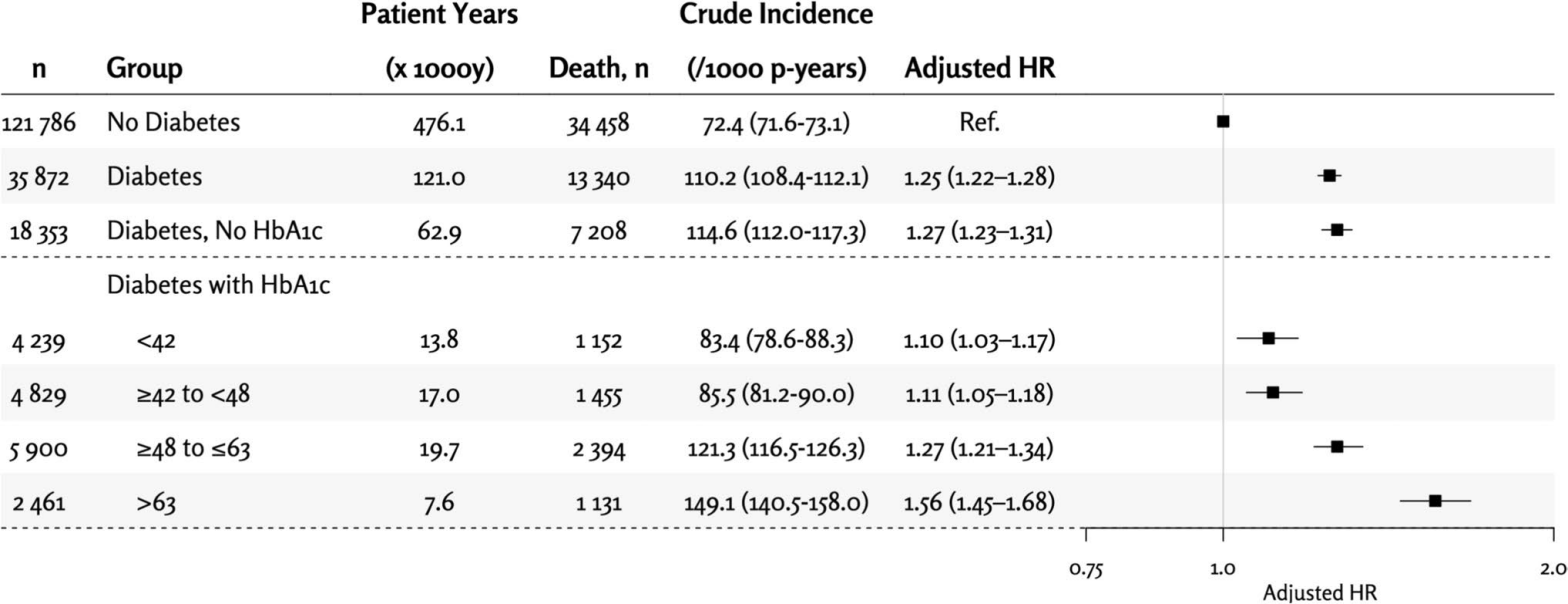
Mortality rates and adjusted hazard ratios (HR) among atrial fibrillation patients with and without diabetes mellitus, categorized by diabetes status and baseline HbA1c (mmol/mol) levels

**Fig. 3:**
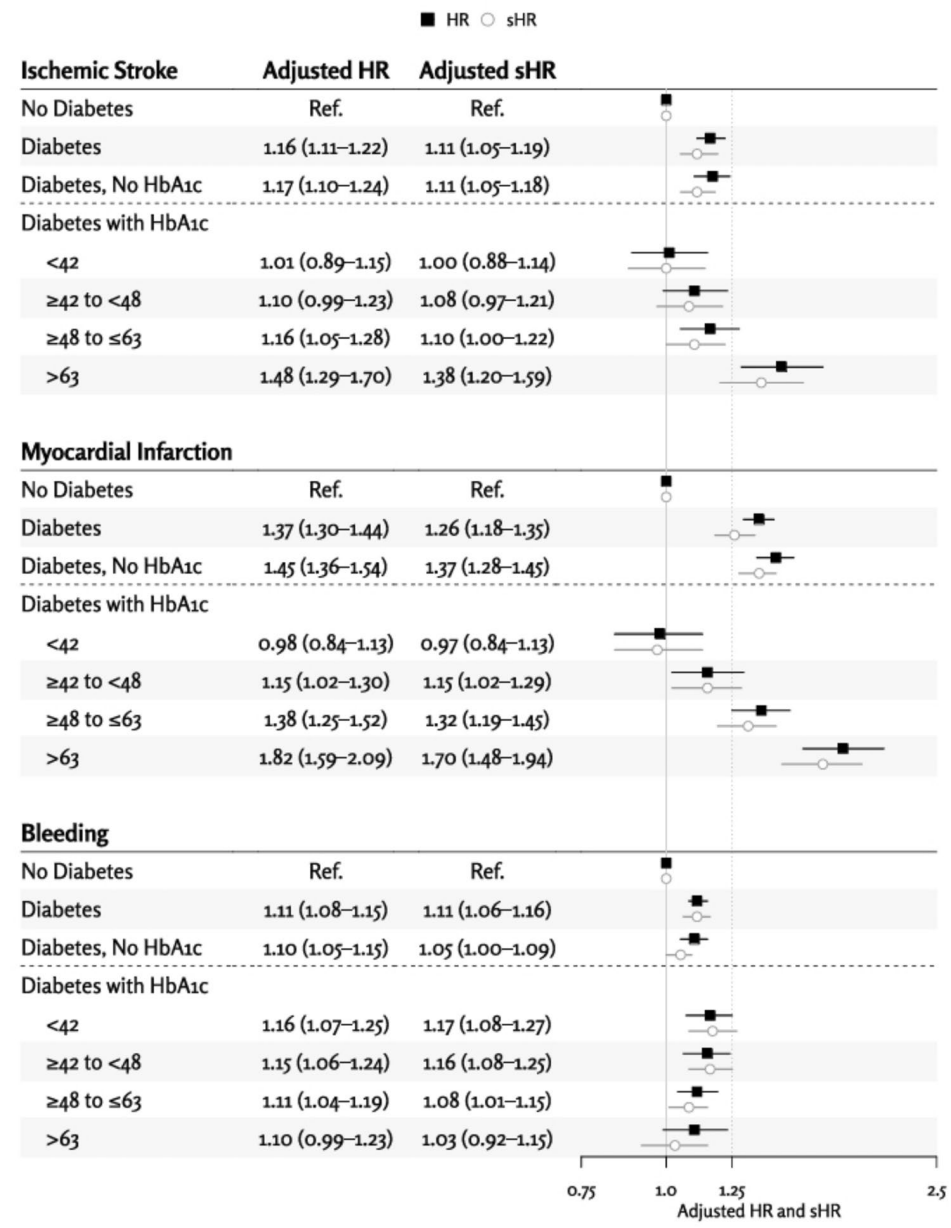
Adjusted hazard ratios and subdistribution hazard ratios of ischemic stroke, myocardial infarction, and bleeding events among atrial fibrillation patients with and without diabetes mellitus, categorized by diabetes status and baseline HbA1c (mmol/mol) levels

Patients with DM experienced higher crude incidence rates across all evaluated outcomes, and the presence of DM associated with significantly increased adjusted HRs and sHRs for death, IS, MI, and bleeding when compared to patients without DM. The HRs and sHRs for all outcomes in the subgroup of patients without baseline HbA1c were in the same range as the HbA1c 48–63 mmol/mol subgroup (Figs. 2 and 3). HRs and sHRs were highest for mortality and myocardial infarction. Among patients with DM, elevated baseline HbA1c levels were associated with greater hazards of death, IS, and MI. Continuous spline models among patients with DM are presented in Fig. 1.

### Mortality

Among patients with DM, crude mortality rates were comparable between the < 42 mmol/mol and ≥ 42 to < 48 mmol/mol subgroups but increased progressively across all higher HbA1c subgroups. The adjusted HRs for death were elevated across all HbA1c subgroups, with a similar progressive increase as seen in the crude incidence rates (Fig. 2). Continuous spline modeling among patients with DM (reference: 48 mmol/mol) revealed a visual J-shaped association. The relative hazard for mortality reached its nadir at approximately 40–42 mmol/mol. Although the point estimate rose at HbA1c levels below this, the increase was not statistically significant (Fig. 1).

### Ischemic stroke and myocardial infarction

Crude incidence rates of both IS and MI in patients with DM were progressively higher with increasing HbA1c. The smallest increments were observed between the lowest and second-lowest HbA1c subgroups. In the subgroup of patients with HbA1c < 42 mmol/mol, the adjusted HRs and sHRs for IS and MI were nearly identical to the reference group of AF patients without DM, with hazards increasing progressively and substantially in the highest HbA1c subgroup (> 63 mmol/mol) (Fig. 3 and Table 2). Continuous spline modelling demonstrated a visual J-shaped association for both outcomes, with the lowest relative hazard near 40 mmol/mol. Although the point estimate for IS hazard dipped below 1.00, this reduction did not achieve statistical significance. Similarly to the mortality analysis, the rise in hazard below approximately 40 mmol/mol was not statistically significant (Fig. 1).

### Bleeding events

In contrast to ischemic outcomes and mortality, no association between baseline HbA1c level and bleeding hazard was observed. Crude bleeding incidence was high but relatively uniform across all HbA1c subgroups, and compared to patients without DM, HRs and sHRs were elevated but remained stable across the HbA1c spectrum (Fig. 3 and Table 2).

**Table 2:** Crude incidence rates of ischemic stroke, myocardial infarction, and bleeding events among atrial fibrillation patients with and without diabetes mellitus, categorized by diabetes status and baseline HbA1c (mmol/mol) levels

**Group**	**Patient years (× 1000 years)**	**Events, n**	**Crude Incidence (/1000 p-years)**
*Ischemic stroke*			
No Diabetes	460.0	7 528	16.4 (16.0–16.7)
Diabetes	116.5	2 546	21.9 (21.0–22.7)
Diabetes, No HbA1c	60.4	1 347	22.3 (21.1–23.5)
Diabetes with HbA1c			
< 42	13.4	235	17.5 (15.4–19.9)
≥ 42 to < 48	16.4	325	19.8 (17.7–22.1)
≥ 48 to ≤ 63	19.0	432	22.7 (20.7–25.0)
> 63	7.2	207	28.6 (24.8–32.8)
*Myocardial infarction*			
No Diabetes	466.3	5 316	11.4 (11.1–11.7)
Diabetes	117.0	2 529	21.6 (20.8–22.5)
Diabetes, No HbA1c	60.6	1 381	22.8 (21.6–24.0)
Diabetes with HbA1c			
< 42	13.5	185	13.7 (11.8–15.8)
≥ 42 to < 48	16.5	286	17.3 (15.3–19.4)
≥ 48 to ≤ 63	19.0	447	23.5 (21.4–25.8)
> 63	7.3	230	31.7 (27.7–36.1)
*Bleeding*			
No Diabetes	443.4	15 115	34.1 (33.5–34.6)
Diabetes	110.9	5 267	47.5 (46.2–48.8)
Diabetes, No HbA1c	60.6	2 653	46.0 (44.3–47.8)
Diabetes with HbA1c			
< 42	12.6	629	49.8 (46.0–53.8)
≥ 42 to < 48	15.4	752	48.4 (45.0–52.0)
≥ 48 to ≤ 63	18.0	894	49.6 (46.4–52.9)
> 63	7.0	339	48.4 (43.4–53.9)

## Discussion

This nationwide retrospective cohort study of patients with incident AF demonstrated a strong association between DM and an increased hazard of mortality, IS, MI, and bleeding. Spline analysis exhibited a non-linear, visually observed J-shaped association between HbA1c levels and mortality, IS, and MI, primarily driven by significantly increasing hazards towards higher HbA1c levels. The hazard for bleeding remained relatively stable across the HbA1c spectrum. While patients with DM and HbA1c < 42 mmol/mol exhibited HRs for ischemic events that were not statistically different from patients without DM in categorical analyses, these findings must be interpreted with caution given the observational nature of the study, unmeasured residual confounding and overall high mortality in this population.

Previous registry studies have established that DM is associated with a poorer prognosis in patients with AF, and the progressive association between elevated HbA1c levels and increased mortality and IS has also been reported [6–10, 18, 19]. Our observation of a sharp increase in cardiovascular events and mortality at higher HbA1c levels (> 63 mmol/mol) aligns with these results. While continuous HbA1c analysis demonstrated non-significant trends towards increased hazards of mortality, IS and MI at the lower end of the HbA1c spectrum (< 40–42 mmol/mol), it is important to note that our sensitivity analyses demonstrated that the magnitude of this increase was sensitive to spline knot placement. The visually apparent increased hazard at low HbA1c must be interpreted cautiously due to sparse data and possible residual confounding. This non-significant rise in mortality aligns with previous observations in elderly cohorts of patients with DM. Intensive glucose lowering therapies as well as low HbA1c levels have been associated with increased mortality in elderly individuals with DM, a finding likely driven by a higher susceptibility to severe hypoglycemia and the compounding effects of frailty, malnutrition or multi-morbidity [20–23]. Most guidelines recommend individualized, less stringent treatment goals for older patients, particularly those with frailty or multi-morbidity [3, 23, 24]**.**

Although DM is associated with bleeding in AF populations, neither continuous nor categorical HbA1c analyses showed a significant association between HbA1c and bleeding hazard [25, 26]. The higher bleeding hazard in our DM population was likely related to the higher prevalence of comorbidities in patients with DM (particularly hypertension, impaired renal function, and a history of bleeding) and may have been further associated with the use of antithrombotic and anticoagulant drugs as well as other factors [27]. The hazard of bleeding, however, remained higher after adjustment for age, hypertension, CKD, previous bleeding, and anticoagulant use.

The relative hazards for adverse outcomes among patients with DM lacking a baseline HbA1c measurement closely mirrored those of the overall diabetic cohort. The baseline variables of these non-HbA1c patients were very similar to the patients with baseline HbA1c, validating the generalizability of our results. The only clear difference was the higher prevalence of hyperlipidemia and statin use in the group with known HbA1c. This may be explained by the fact that hyperlipidemia was partly defined based on laboratory values (Supplementary Table ST1), and that patients with known HbA1c were also more likely to have undergone additional laboratory testing.

It is noteworthy that this study was conducted primarily during a period when cardioprotective DM medications such as sodium-glucose co-transporter-2 (SGLT2) inhibitors and glucagon-like peptide-1 (GLP-1) receptor agonists had only recently emerged [28, 29]. Since these therapies offer cardiovascular protection in addition to HbA1c reduction, it would be of clinical interest to investigate whether their widespread use has mitigated the cardiovascular morbidity and mortality observed in our study.

### Study strengths

While several large registry-based studies have provided valuable insights into the relationship between AF and DM, a key strength of the FinACAF cohort is its comprehensive capture of patients across all levels of care within a highly validated national healthcare registry system, further strengthened by the linkage of individual laboratory data [11, 30]**.** Previous registry-based studies on AF patients may be more susceptible to selection and information biases due to the reliance on data from limited care settings.

### Study limitations

Our data are based on administrative records entered during routine clinical practice, which introduces inherent limitations related to data accuracy and completeness, as well as residual confounding. We lacked data on important clinical variables such as body mass index, smoking status, DM duration, and frailty. Since factors such as obesity and smoking are associated with poor glycemic control and adverse cardiovascular events, their absence may result in an overestimation of the direct association between HbA1c levels and the studied outcomes. Additionally, a longer duration of DM represents cumulative vascular damage, acting as an unmeasured confounder associated with adverse outcomes [9]. Frailty in elderly patients can significantly alter both mortality risk and glycemic targets, potentially confounding our observations [23]. The severity of bleeding events is challenging to assess accurately when relying solely on registry data. We were also unable to reliably distinguish between type 1 and type 2 DM, which constitutes a clinically important limitation, given that susceptibility to severe hypoglycemia, cardiovascular phenotype, and overall cardiovascular risk differ markedly between these populations [31]. Finally, owing to laboratory data coverage representing 77% of the Finnish population, a substantial proportion of patients with DM could not be directly included in our HbA1c analysis.

## Conclusion

DM at the time of initial AF diagnosis was significantly associated with increased mortality and elevated rates of IS, MI, and bleeding compared to patients without DM. Continuous HbA1c modeling revealed an association of increased HRs for mortality and ischemic events in patients with higher HbA1c levels.

## Conflict of interest

The authors declare the following financial interests/personal relationships which may be considered as potential competing interests; Elis Kouki: None declared. Birgitta Salmela: None declared. Olli Halminen: None declared. Aapo Aro: Grants: Finnish foundation for Cardiovascular Research, Sigrid Juselius Foundation; Speaker: Abbot, Johnson and Johnson, Sanofi, Bayer, Boehringer-Ingelheim. Konsta Teppo: Grants: The Finnish Foundation for Cardiovascular Research, Aarne and Aili Turunen Foundation. Leo Niskanen: Speaker: Amgen, Boehringer-Ingelheim, NovoNordisk, Sanofi, MSD, AstraZeneca, Eli Lilly; research support from NovoNordisk to the hospital; scientific advisory boards Eli Lilly, Amgen, Boehringer-Ingelheim, AstraZeneca, MSD, NovoNordisk. Jari Haukka: Consultant: Research Janssen RandD; Speaker: Bayer Finland. Research Grants: Jane and Aatos Erkko Foundation, Avohoidon Tutkimussäätiö, European Commission (Brussels, 282526). Jukka Putaala: Speaker: Bayer, Boehringer-Ingelheim, BMS-Pfizer, Abbott; Advisory board: Novo Nordisk, Herantis Pharma; Visiting editor: Terve Media; Stock ownership: Vital Signum. Miika Linna: Speaker: BMS-Pfizer-alliance, Bayer, Boehringer-Ingelheim. Pirjo Mustonen: Consultant: Roche, BMS-Pfizer-alliance, Novartis Finland, Boehringer-Ingelheim, MSD Finland. Juha Hartikainen: Research grants: The Finnish Foundation for Cardiovascular Research, EU Horizon 2020, EU FP7; Advisory Board Member: BMS-Pfizer-alliance, Novo Nordisk, Amgen; Speaker: Cardiome, Bayer. K.E. Juhani Airaksinen: Grants: The Finnish Foundation for Cardiovascular Research; Speaker: Bayer, Pfizer, and Boehringer-Ingelheim; Advisory board: Bayer, Pfizer and AstraZeneca. Mika Lehto: Consultant: BMS-Pfizer-alliance, Bayer, Boehringer-Ingelheim; Speaker: BMS-Pfizer-alliance, Bayer, Boehringer Ingelheim, Terve Media, and Orion Pharma. Research grants: Aarne Koskelo Foundation, The Finnish Foundation for Cardiovascular Research, Yrjö Jahnsson Foundation, Sigrid Juselius Foundation. and Helsinki and Uusimaa Hospital District research fund.

## Informed consent

This study is based on retrospective register data. Thus, no patient contacts were involved in any phase of the study, and no patient consent was required according to Finnish legislation. The study conforms to the Declaration of Helsinki as revised in 2013 and to European General Data Protection Regulation (EGDPR).

## Supplementary Information

Below is the link to the electronic supplementary material.

Supplementary file1 (DOCX 640 KB)

## Data Availability

Data may be obtained from a third party and are not publicly available. Due to the sensitive nature of the data collected for this study, requests to access the data set from qualified researchers trained in human subject confidentiality protocols may be sent to the Finnish national registry holders (KELA, Finnish Institute for Health and Welfare, Population Registry Center and Tax Registry) through Findata (https://findata.fi/en/).
