# Supplementary material for: Glycemic control and clinical outcomes in patients with diabetes and atrial fibrillation: a nationwide cohort study
Source: Acta Diabetol. 2026 Jun 22;63(8):1459–68. doi: 10.1007/s00592-026-02725-1 (PMC13469207; doi:10.1007/s00592-026-02725-1)
Supplement: Supplementary file 1 [file 592_2026_2725_MOESM1_ESM.docx]

**Supplementary Figure SF1: Patient enrollment and exclusion criteria**

**Supplementary Figure SF2: Sensitivity analysis of spine knot placements**

Top row: 5-knots placed at 5^th^, 27.5^th^, 50^th^, 72.5^th^, and 95^th^ percentiles.
Bottom row: 4-knots places at 5^th^, 35^th^, 65^th^, and 96^th^ percentiles.

**Supplementary table ST1: Definitions of baseline variables and comorbidities**

|  | **ICD-10** | **ICPC-2** | **Reimburse-ment code** | **ATC-Code** | **Laboratory** |
| --- | --- | --- | --- | --- | --- |
| **Hyperlipidemia** | E78 | T93 | 206 | C10 | Chol > 5 mmol/l Chol-LDL > 3 mmol/l |
| **Hypertension** | I10-I15 | K85  K86  K87 | 205 | C03A, C03B, C03DB, C03EA, C07A, C08CA, C08D, C09 |  |
| **Diabetes** | E10-E14 | T89 T90 | 103 215 | A10 | B-HbA1c ≥ 48 mmol/mol |
| **Ischemic stroke** | I63-I64  I69.3-I69.8 | K90 |  |  |  |
| **Transient ischemic attack** | G45 | K89 |  |  |  |
| **Heart failure** | I50, I11.0, I13.0, I13.2 | K77 | 201 |  |  |
| **Vascular disease** | I20-I25  I65-I66  I67.2  I70 | K74  K75  K76  K91  K92 | 206 |  |  |
| **Coronary artery disease** | I20-I25, Z95.1 |  |  |  |  |
| **Myocardial infarction** | I21-I22 |  |  |  |  |
| **Pulmonary embolism** | I26 |  |  |  |  |
| **Venous thromboembolism** | I80-I83 |  |  |  |  |
| **Cardiomyopathy** | I42.0-I42.2  I42.9 |  |  |  |  |
| **Cancer** | National cancer register |  |  |  |  |
| **Thyrotoxicosis** | E05 |  |  |  |  |
| **Sleep apnea** | G47.3 |  |  |  |  |
| **COPD** | J44 |  |  |  |  |
| **Dementia** | F00-F03, G30 |  |  |  |  |
| **Psychiatric disease** | F04-F99 |  |  |  |  |
| **Bleeding history** | D50.0, D62, D68.3, I60-I62, I69.0-I69.2, I85.0, I86.4, J94.2, K22.1, K22.3, K22.6, K25.0, K25.2, K25.4, K25.6, K26.0, K26.2, K26.4, K26.6, K27.0, K27.2, K27.4, K27.6, K28.0, K28.2, K28.4, K28.6, K29.0, K62.5, K63.1, K63.3, K92.0-K92.2, N02, R04, R31, R58, S06.2-S06.6, S06.8 |  |  |  |  |
| **Alcohol use disorder** | F10 |  |  |  |  |
| **Impaired renal function** | N18, Z49 |  |  |  |  |
| **Liver cirrhosis or failure** | K70.2-K70.4, K71.7, K71.8, K72, K74 |  |  |  |  |
| **Use of statin** |  |  |  | C10AA, C10BA, C10BX |  |
| **Use of other Lipid lowering medication** |  |  |  | C10AB, C10AC, C10AX09, X10BA02, C10BA05, C10BA06, C10BA10, C10BA11, C10AX13, X10AX14 |  |
| **CHA2DS2-VA score (0-8 points)** | Congestive heart failure (1 point), Hypertension (1 point), Age ≥ 75 years (2 points), Diabetes (1 point), previous Stroke or TIA (2 points), vascular disease (1 point), Age 65-74 years (1 point) | | | |  |
| **HAS-BLED score** | Hypertension, Abnormal renal/liver function (1 point each), Stroke, Bleeding history or predisposition, Labile INR, Elderly (>65 years), Drugs/alcohol concomitantly (1 point each). | | | |  |

Abbreviations: ATC, anatomic therapeutic chemical; ICD-10, International Classification of Diseases, Tenth Revision; ICPC-2, International Classification of Primary Care, Second Edition

Comorbidities were obtained using data hierarchically as follows:

1) ICD-10 codes from Finnish Care Register (HILMO)

2) ICD-10 codes from Finnish Care Register (AvoHILMO)

3) International Classification of Primary Care, 2^nd^ edition (ICPC-2) codes from Finnish Care Register (AvoHILMO)

4) entitlement to reimbursement for the comorbidity or disease from KELA

5) prescribed medication (ATC codes) from KELA

- hypertension:
  - C03A, C03B, C03DB, C03EA and C08CA can also be used after the diagnosis of atrial fibrillation (AF)
  - beta blockers (C07) and diltiazem or verapamil (C08D) algorithm for diagnosis of hypertension:
    - can be used at baseline only, i.e. before the diagnosis of AF
    - cannot be utilized after the diagnosis of AF, because these are used in the treatment of AF
    - If a patient has a diagnosis of coronary artery disease (I20-I25) then beta blockers, diltiazem or verapamil cannot be utilized for diagnosing hypertension
  - drugs acting on the renin angiotensin aldosterone system (C09) for the diagnosis of hypertension can also be used after the diagnosis of AF
  - if a patient has the diagnosis of heart failure (I50) and/or cardiomyopathy (I420-I422, I429) then C09 cannot be utilized for diagnosing hypertension
- C09 cannot provide a diagnosis of heart failure, i.e. utilization of this drug group is limited to diagnosis of hypertension

Baseline medication was defined as purchase of a medication up to one year before the cohort entry date.

**Supplementary table ST2: Definitions of outcomes**

| **Variable** | **Definition (ICD-10)** |
| --- | --- |
| Ischemic stroke | I63-I64, I693-I698 |
| Myocardial infarction | I21-I22 |
| Bleeding events | D500, D62, D683, I60-I62, I690-I692, I850, I864, J942, K221, K223, K226, K250, K252, K254, K256, K260, K262, K264, K266, K270, K272, K274, K276, K280, K282, K284, K286, K290, K625, K631, K633, K920-K922, N02, R04, R31, R58, S062-S066, S068 |

Abbreviations: ICD-10, International Classification of Diseases, Tenth Revision

**Supplementary table ST3: Baseline variables of patients with diabetes mellitus stratified by baseline HbA1c availability**

|  | **DM,  no HbA1c**  **n = 18 353** | **DM  with HbA1c n = 17 519** | **P <0.05  * = yes** | **Unweighted SMD** | **Weighted SMD** |
| --- | --- | --- | --- | --- | --- |
| **Demographics** |  |  |  |  |  |
| Sex, Female (%) | 48.0 | 47.3 |  | 0.015 | 0.000 |
| Mean age, years (SD) | 74.9 (11.0) | 74.7 (10.1) |  | 0.016 | 0.000 |
| **Income quartiles (%)** |  |  | * | 0.087 | 0.000 |
| 1st (lowest) | 31.9 | 28.2 |  |  |  |
| 2^nd^ | 27.0 | 27.8 |  |  |  |
| 3^rd^ | 22.7 | 25.1 |  |  |  |
| 4th (highest) | 18.3 | 19.0 |  |  |  |
| **Education (%)** |  |  | * | 0.061 | 0.000 |
| 1st (lowest) | 58.9 | 56.9 |  |  |  |
| 2^nd^ | 26.5 | 26.4 |  |  |  |
| 3rd (highest) | 14.5 | 16.7 |  |  |  |
| **Comorbidities (%)** |  |  |  |  |  |
| Hypertension | 90.3 | 91.9 | * | 0.058 | 0.001 |
| Heart failure | 25.0 | 24.1 |  | 0.020 | 0.000 |
| Hyperlipidemia | 70.7 | 76.4 | * | 0.129 | 0.000 |
| Ischemic stroke or TIA | 14.1 | 14.1 |  | 0.007 | 0.003 |
| Vascular disease | 38.9 | 41.1 | * | 0.045 | 0.000 |
| Coronary artery disease | 32.0 | 33.7 | * | 0.036 | 0.005 |
| Myocardial infarction | 12.7 | 12.7 |  | 0.001 | 0.017 |
| Thyrotoxicosis | 1.1 | 0.9 |  | 0.016 | 0.015 |
| Cardiomyopathy | 2.1 | 2.0 |  | 0.008 | 0.006 |
| Venous thromboembolism | 6.2 | 6.6 |  | 0.016 | 0.015 |
| Pulmonary embolism | 1.6 | 1.6 |  | 0.005 | 0.005 |
| Prior bleeding | 13.8 | 14.2 |  | 0.013 | 0.000 |
| Alcohol use disorder | 4.6 | 5.0 |  | 0.019 | 0.000 |
| COPD | 6.7 | 6.8 |  | 0.004 | 0.002 |
| Sleep apnea | 6.5 | 8.4 | * | 0.071 | 0.056 |
| Cancer | 21.9 | 23.7 | * | 0.042 | 0.000 |
| Dementia | 6.6 | 5.9 | * | 0.029 | 0.000 |
| Psychiatric disorder | 15.2 | 17.2 | * | 0.056 | 0.000 |
| Liver cirrhosis or failure | 0.8 | 1.1 |  | 0.029 | 0.001 |
| Impaired renal function | 7.4 | 8.8 | * | 0.050 | 0.000 |
| **Risk scores** |  |  |  |  |  |
| Mean CHA2DS2-VA score (SD) | 4.2 (1.5) | 4.3 (1.5) |  | 0.013 | 0.005 |
| Mean modified HAS-BLED score (SD) | 2.4 (1.0) | 2.5 (1.0) | * | 0.079 | 0.027 |
| **Medication (%)** |  |  |  |  |  |
| Statin | 53.3 | 61.5 | * | 0.167 | 0.000 |
| Lipid lowering medication, other | 2.2 | 2.9 | * | 0.045 | 0.030 |

SMD: Standardized mean difference
DM;Diabetes mellitus.
CHA2DS2-VA (congestive heart failure, hypertension, age ≥75 years, diabetes, history of stroke/TIA, vascular disease, age 65-74 years).
HAS-BLED (hypertension, abnormal renal or liver function, prior stroke, bleeding history, age >65 years, alcohol abuse, concomitant antiplatelet/nonsteroidal anti-inflammatory drugs (no labile INR available, max score 8).

**Supplementary Table ST4 Baseline variables of the study cohort**

|  | **No DM** | **DM with HbA1c (n=17 519, 11.1 %)**  **HbA1c (mmol/mol)** | | | |
| --- | --- | --- | --- | --- | --- |
|  |  | **<42** | **≥42 to <48** | **≥48 to ≤63** | **>63** |
| **N (% of total cohort)** | 121 786 (77.2) | 4 329 (2.7) | 4 829 (3.1) | 5 900 (3.7) | 2 461 (1.6) |
| **Income quartiles**^*,**^ |  |  |  |  |  |
| 1st (lowest) | 24.6 | 22.8 | 26.7 | 31.1 | 33.5 |
| 2nd | 23.4 | 27.1 | 28.5 | 28.2 | 26.4 |
| 3rd | 25.7 | 27.2 | 25.3 | 23.9 | 23.6 |
| 4th (highest) | 26.4 | 22.9 | 19.6 | 16.9 | 16.4 |
| **Education**^*,**^ |  |  |  |  |  |
| 1st (lowest) | 49.4 | 52.1 | 56.9 | 60.4 | 57.0 |
| 2nd | 28.4 | 28.2 | 26.0 | 24.4 | 29.0 |
| 3rd (highest) | 22.2 | 19.7 | 17.1 | 15.3 | 14.0 |
| **Comorbidities** |  |  |  |  |  |
| Thyrotoxicosis | 1.0 | 1.0 | 0.9 | 1.1 | 0.6 |
| Cardiomyopathy^*, **^ | 1.7 | 1.8 | 1.6 | 2.1 | 3.0 |
| Venous thromboembolism^*^ | 5.7 | 6.3 | 6.4 | 6.9 | 6.9 |
| Pulmonary embolism^*^ | 1.3 | 1.5 | 1.5 | 1.5 | 2.1 |
| Alcohol use disorder^*,**^ | 4.2 | 7.2 | 3.8 | 3.9 | 6.1 |
| COPD^*^ | 4.7 | 6.1 | 6.7 | 7.1 | 7.3 |
| Sleep apnea^*,**^ | 3.0 | 8.6 | 7.8 | 7.9 | 10.1 |
| Cancer^*,**^ | 20.5 | 24.3 | 23.6 | 24.3 | 21.1 |
| Dementia^*,**^ | 5.0 | 5.2 | 5.3 | 6.3 | 7.3 |
| Psychiatric disorder^*,**^ | 13.8 | 20.3 | 14.8 | 16.0 | 19.7 |

*p<0.05 DM vs. Non-DM; **p<0.05 within DM HbA1c subgroups
DM; Diabetes mellitus.
CHA2DS2-VA (congestive heart failure, hypertension, age ≥75 years, diabetes, history of stroke/TIA, vascular disease, age 65-74 years).
HAS-BLED (hypertension, abnormal renal or liver function, prior stroke, bleeding history, age >65 years, alcohol abuse, concomitant antiplatelet/nonsteroidal anti-inflammatory drugs (max score 8 as labile INR was unavailable).
